# Esophageal perforation in closed neck trauma

**DOI:** 10.5935/1808-8694.20130022

**Published:** 2015-10-14

**Authors:** Agnaldo José Graciano, Adrian Maurício Stockler Schner, Carlos Augusto Fischer

**Affiliations:** MD, MSc in Sciences (Otorhinolaryngologist and Head and Neck Surgeon - Hospital São José e Centro Hospitalar Unimed - Joinville - SC); MD (Chest Surgeon - Hospital São José e Centro Hospitalar Unimed - Joinville - SC); MD (Head and Neck Surgeon and Maxillo-Facial Surgeon - Hospital São José e Centro Hospitalar Unimed - Joinville - SC). Centro Hospitalar Unimed - Joinville - Santa Catarina

**Keywords:** blunt wounds, esophageal perforation, neck injuries

## INTRODUCTION

Esophageal perforation is rare in blunt neck injuries, but it carries a 20% mortality rate. This high mortality rate is caused by delays in diagnosis because of not considering the injury in these circumstances, preventing proper treatment to be carried out before severe complications ensue.

Here, we provide an example and the basis for its recognition and management, according to data from the current literature.

## CASE PRESENTATION

**Figure 1:**
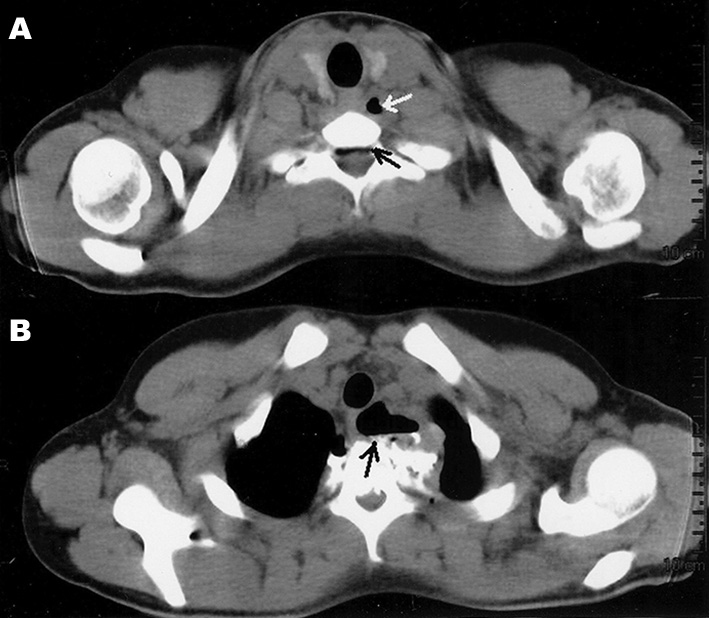
A: CT scan showing neck extraluminal air (clear arrow) and air in the spinal canal (dark arrow). B: Extensive air collection

A 17 year-old male came to our clinic complaining of pain and vomits upon swallowing for about one week after having fell from a bicycle and having his body turned on its neck axis. He was assessed twice in other clinics, and was prescribed analgesics and anti-vomiting medication, without improvements. He was submitted to a CT scan, which showed neck emphysema and pneumorachis (Figure 1 A-B), and an MRI scan confirmed he also had a ligament injury between T1 and T2 and a blood workup showed mild leukocytosis. Although the gastric endoscopy did not show injuries, immediate exploratory neck surgery was indicated, which revealed a 1.5 cm esophageal rupture in the neck-chest transition area, and rupture of muscles and intervertebral ligaments. The esophagus was sutured and the neck was drained. The patient was submitted to antibiotic treatment and parenteral nutrition with resolution of the perforation, without other complications.

## DISCUSSION

Esophageal perforations have been increasingly seen because of a raise in the number of diagnostic and treatment endoscopies, and its incidence has been estimated to be 3.1:1000,000/year^1^. Today, 60% of the perforations are iatrogenic, secondary to endoscopic and neck/chest procedures, 15% are spontaneous (Boerhaave's syndrome), more commonly seen in the thoracic or abdominal portions of the esophagus, and only 2% to 15% are caused by neck trauma. Most of the trauma-induced esophagus injuries are located in the neck (57%), followed by the its thoracic and abdominal portions, caused by firearm (78.8%) or blunt weapons (18.5%) and only 2.7% are associated with blunt neck traumas^2^.

In blunt neck injuries, it is important to suspect of esophageal perforations in situations involving fast acceleration and deceleration associated with neck hyperex-tension with the esophagus being pushed against the spine.

The most common symptom is ody-nophagia, complained by 70% to 92% of the patients. Special attention must be given to pain, vomit and dyspnea upon deglutition (Mackler's triad) - in 25% of the patients. Although not very specific for esophageal injuries, thoracic and/or neck subcutaneous emphysema and pneumorachis (air in the spinal canal) hints at the possibility of rupture of some structure which had air^3^.

When there is a suspicion of esophageal perforation, complementary tests must be carried out immediately, because there is an increase in the occurrence of severe complications when the investigation and treatment happen 24 hours after the injury and, after such time, mortality may reach up to 60% in cases of late treatment.

The test-of-choice is esophagography, yielding between 90% and 100% sensitiveness. Water-soluble contrasts (Gastrografin^®^) are preferable, due to a lesser risk of mediasti-nal inflammatory reaction, more commonly associated with the use of barium contrasts. Nonetheless, its use does not detect small neck esophageal perforations in up to 50% of the cases, favoring the use of barium contrast medium in some institutions^4^. Stiff esophagoscopy was the second most utilized exam in a study involving 405 patients from 34 trauma centers in the US, followed by flexible endoscopy -which may be associated with esophagography in cases of suspicion of esophageal injury with an initially-normal radiographic exam, boasting up to 100% specificity^5^.

The most recommended initial approach is primary esophageal suture, utilized in about 80% of the cases, associated with neck drainage, antibiotic treatment and enteral or parenteral nutrition^6^.

## FINAL REMARKS

Esophageal perforation is rare in blunt neck injuries, but it should be suspected in high-speed injuries with neck over-extension.
